# Histological, Histochemical, and Protein Changes after Induced Malocclusion by Occlusion Alteration of Wistar Rats

**DOI:** 10.1155/2014/563463

**Published:** 2014-06-17

**Authors:** Carolina de Souza Guerra, Yamba Carla Lara Pereira, João Paulo Mardegan Issa, Kelly Galisteu Luiz, Elaine A. Del Bel Guimarães, Raquel Fernanda Gerlach, Mamie Mizusaki Iyomasa

**Affiliations:** Department of Morphology, Physiology and Basic Pathology, Ribeirão Preto School of Dentistry, University of São Paulo, FORP/USP, P.O. Box 14040-904, Ribeirao Preto, SP, Brazil

## Abstract

Although disorders of the stomatognathic system are common, the mechanisms involved are unknown. Our objective was to study the changes in the masseter muscles after unilateral exodontia. Molar extraction was performed on Wistar rats (left side), and the animals were sacrificed after either 14 or 26 days. The masseter muscle was processed for histological analysis, conventional and *in situ* zymography, and immunohistochemistry. The morphological analysis showed unique and specific characteristics for the experimental group. By conventional zymography no significant values of 72 kDa MMP-2 (*P* < 0.05) were found in both of the sides of masseter muscle after 14 and 26 days of unilateral extraction. The *in situ* zymography showed gelatinolytic activity on all deep masseter muscles, with significant increase on the contralateral side after 14 and 26 days (*P* < 0.05). The immunohistochemistry demonstrated greater expression of MMP-2 than MMP-9 and MMP-14 in all masseter muscles and there were few differences in the staining of 4 TIMPs. This knowledge about morphology and molecular masticatory muscle remodeling following environmental interventions can be used to develop clinically successful treatments.

## 1. Introduction

The stomatognathic system is highly complex and composed of several interrelated structures. Among the structures of the stomatognathic system, the role of muscles in the etiology of headaches [1], facial pain [2], the influence of muscles in the etiology of some facial deformity, and on treatment outcome [3] has aroused interest among researchers and clinicians alteration. However in dentistry, the mechanisms of masticatory muscles remodeling after orthopedic or surgical interventions are still poorly understood, by this way information could help in the prevention of relapse or treatment failure [4].

It is known that extracellular matrix (ECM) placed in tendon tissue as well as peri and intramuscularly ensures a functional link between the skeletal muscle cell and the bone [5], however, search about ECM response to mechanical loading and its function on masticatory muscle adaptation are scarce. The ECM is a conglomerate of substances, in which histochemical and biophysical properties allow for the construction of a flexible network that integrates information from loading and converts it into mechanical capacities [6].

The connective tissue of skeletal muscle then seems to be a key element involved in the remodeling of the masticatory muscle during functional appliance therapy or developmental situations. Some studies in the nonorthodontic literature have shown that the matrix metalloproteinases (MMPs) are involved in pathological and physiological processes of the skeletal muscles remodeling [7, 8]. The MMPs are initially synthesized in an enzymatically inactive or zymogen form [9] and are activated in some conditions. They are widely distributed in craniofacial tissues [10] such as oral mucosa [11] gingiva [12, 13], tooth buds [10], and forming enamel [14, 15]. It is also known that the tissue inhibitors metalloproteinases (TIMPs) are synthesized to bind directly to active enzymes to prevent their activity [16]. In human masseter muscle, Tippett et al. [17] found that an excess of tissue inhibitors metalloproteinase (TIMP-1) restricted extracellular matrix turnover and is interrelated with MMP-2 and MMP-9.

The present study investigates the hypothesis that MMPs and TIMPs expressions and histological characteristics on masseter muscle were altered after unilateral exodontia. To understand the mechanisms involved in the masticatory muscle remodeling process, we performed extraction of the upper molars on the left side to examine how its interventions affect the masseter muscles.

## 2. Material and Methods

### 2.1. Animals

Thirty young male Wistar rats weighing 200 g at the beginning of the procedures were randomly distributed into two groups: control (*n* = 10) and experimental (*n* = 20). In the experimental group, 10 animals were sacrificed after 14 days and 10 were sacrificed after 26 days. The animals were fed with a standard diet and water* ad libitum*. Polyethylene cages with a maximum of four animals were maintained in a controlled temperature room (23°C to 25°C) with light and dark cycles of 12 h. All procedures of this study were approved by the Local Ethics Committee on the Use of Animals according to the internal laws for animals use, protocol number 08.1.290.53.9.

### 2.2. Induction of Occlusal Alteration in Animals

In the experimental group, the unilateral exodontia was performed by extraction of the upper molars on the left side. The control group had no buildups and received only a sham operation (this group was subjected to the same surgical stress during mandible opening of the surgical treatment). Under aseptic conditions, all animals in the control and experimental groups received intraperitoneal anesthesia (tribromoethanol, 0.25 g/kg of body weight). As a prophylactic measure, an antibiotic (penicillin, 24000 IU/kg of body weight, Pentabiotic, Fort Dodge) and an anti-inflammatory (diclofenac sodium, 1 mL/kg of body weight, Voltaren) were administered to the animals in a single dose.

### 2.3. Histological Analysis

Animals from the experimental groups, after 14 (*n* = 5) and 26 days (*n* = 5), and control (*n* = 5) groups were sacrificed by decapitation after administration of intraperitoneal anesthesia of xylazine (10 mg/kg) and ketamine (70 mg/kg). The deep masseter muscle bundles from each side (right and left) were dissected, and the middle portion was snap-frozen in isopentane cooled by liquid nitrogen (−150°C) and kept at −80°C until use. Serial cross sections were cut to a thickness of 10 *μ*m at −20°C using a Leica cryostat microtome and were stained for histological studies. The serial cross sections were stained by hematoxylin-eosin for morphological study, conventional zymography,* in situ* zymography, and immunohistochemistry.

### 2.4. Zymography

Samples of the deep masseter muscle bundle, of each side (right and left), from both the 14- (*n* = 5) and 26- (*n* = 5) day experimental groups and control (*n* = 5) were frozen in dry ice and stored at 80°C until use. These samples were cut into pieces and homogenized for protein extraction using the Bradford method (Sigma) in Tris-CaCl_2_ buffer (Tris 50 mM, pH 7.4, and 10 mM CaCl_2_). Forty micrograms of protein from muscles was run on precast 12% SDS-polyacrylamide gels (PAGE) containing gelatin [18, 19] for electrophoresis analyses. The samples were separated under nonreducing conditions for gelatin-substrate zymography. After electrophoresis, the gel was incubated for 1 h at room temperature in a 2% Triton X-100 solution and then incubated at 37°C for 16 h in Tris-HCl buffer, pH 7.4, containing 10 mMol/L CaCl_2_. The gels were stained with 0.05% Coomassie Brilliant Blue G-250 and then destained with 30% methanol and 10% acetic acid. Gelatinolytic activities were detected as unstained bands relative to the background of Coomassie Blue-stained gelatin. Enzymatic activity was assayed by densitometry using a Kodak Electrophoresis Documentation and Analysis System (EDAS) 290 (Kodak, Rochester, NY). The gel was placed in a solution of methanol and dried for contraction in cellophane. The MMP-2 and MMP-9 proforms were identified as bands at 72 and 92 kDa, respectively, by the relation of log Mr to the relative mobility of Sigma SDS-PAGE LMW marker proteins. MMP activity was further confirmed using the specific inhibitor 10-Phenanthroline (Sigma).

Statistical analysis was done by the Kruskal-Wallis test followed by Dunn's Multiple Comparison test for comparisons between the control and experimental groups (14 and 26 days).

### 2.5. *In Situ* Zymography Using DQ-Gelatin

The DQ-gelatin (DQ-gelatin, E12055; Molecular Probes, Eugene, OR) was dissolved in Tris-CaCl_2_ buffer. Forty microliters were applied to each slide for 60 minutes at room temperature in a dark humid chamber [20]. DQ-gelatin was then gently washed, and the sections were fixed with 4% buffered paraformaldehyde (PFA). The gelatinolytic activity was observed as green fluorescence (absorption maxima, 495 nm; fluorescence emission maxima, 515 nm) by fluorescence microscopy (Fluorescence Microscope Model Axioskop 50 using camera AxioCam MRC; Carl Zeiss, Oberkochen, Germany). Negative control sections were incubated with a metalloproteinase inhibitor (1,10-phenanthroline).

### 2.6. Immunohistochemistry

During the immunohistochemical reactions, sections were first blocked using peroxidase blocking solution for 10 minutes and washed with PBS. They were then incubated with the primary antibody diluted to a final concentration of 1 : 10,000 in PBS for 1 hour at room temperature. Tissue sections were incubated in dark humidified chambers for 1 h with either a mouse anti-MMP-2 (0.1 *μ*g mL^−1^, MAB3308, Chemicon), a mouse anti-MMP-9 (0.1 *μ*g mL^−1^, MAB3309, Chemicon), a mouse anti-MMP-14 (0.1 *μ*g mL^−1^, MAB3317, Chemicon), a mouse anti-TIMP-1 (0.1 *μ*g mL^−1^, MAB3300, Chemicon), a mouse anti-TIMP-2 (0.2 *μ*g mL^−1^, MAB13446, Chemicon), a rabbit anti-TIMP-3 polyclonal antibody (0.1 *μ*g mL^−1^, AB802, Chemicon), or a rabbit anti-TIMP-4 polyclonal antibody (0.1 *μ*g mL^−1^, AB816, Chemicon). After washing with PBS, the sections were incubated with the secondary antibody anti-Mouse poly-HRP for one hour and again washed with PBS. DAB was finally applied and washed with PBS. The DAB slides were then counterstained with hematoxylin, mounted in Entellan, and examined under light microscopy.

## 3. Results

### 3.1. Histological Analysis

#### 3.1.1. Control Group

**Figure 1:**
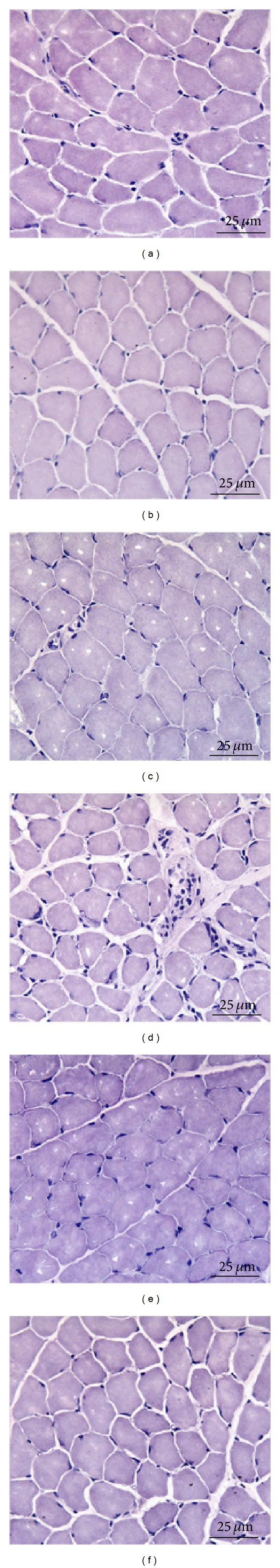
Histological analysis with hematoxylin-eosin staining of the contralateral (a, c, and e) and ipsilateral (b, d, and f) masseter muscle. (a, b) Control group. (c, d) Experimental group 14 days after exodontia. (e, f) Experimental group 26 days after exodontia.

The control group muscles (Figures 1(a) and 1(b)) showed normal characteristics with round nuclei arranged at the periphery, some cells and capillaries in the endomysium, and no morphological difference between the right and left sides.

#### 3.1.2. Fourteen Days: Experimental Group

The muscle ipsilateral to the extracted tooth showed flattened nuclei on the periphery of the fibers (Figure 1(d)). On the contralateral side, fibers were more rounded with some larger nuclei at the periphery, and larger blood vessels were observed in the endomysium (Figure 1(c)).

#### 3.1.3. Twenty Six Days: Experimental Group

The ipsilateral muscle to extracted tooth showed some signs of atrophy and flattened nuclei located on the periphery (Figure 1(f)). Muscle fibers on the contralateral side showed round aspects with a larger diameter (Figure 1(e)).

### 3.2. Zymography

**Figure 2:**
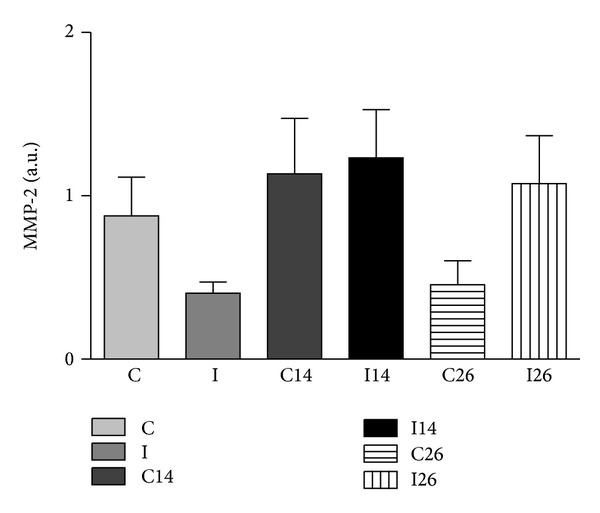
Mean ± SD of the average absorbance values obtained for the 72 kDa MMP-2 protein in masseter muscle zymography gels, represented by the following: C, I contralateral and ipsilateral sides of the control group; C14, I14 contralateral and ipsilateral sides after 14 days of exodontia; C26, I26 contralateral and ipsilateral sides after 26 days of exodontia.

The gel zymography confirmed the presence of gelatinases in all deep masseter muscles of the three groups: control, 14 days, and 26 days after unilateral extraction of upper molar. The 72 kDa and 75 kDa MMP-2 molecular forms are the nonglycosylated and the glycosylated proenzyme forms, respectively, described in the rat. Active MMP-2 (64 kDa) and MMP-9 were not detected.  Figure 2 shows no relevant values (data expressed as the mean absorbance obtained after densitometry quantification) of the MMP-2 in both sides of masseter muscle from the experimental and control groups.

After statistical analysis, tendency was found to increase the amount of 72 kDa MMP-2 in both sides of masseter muscle after 14 days of unilateral extraction, and in the ipsilateral side after 26 days, when compared with the control.

### 3.3. *In Situ* Zymography

**Figure 3:**
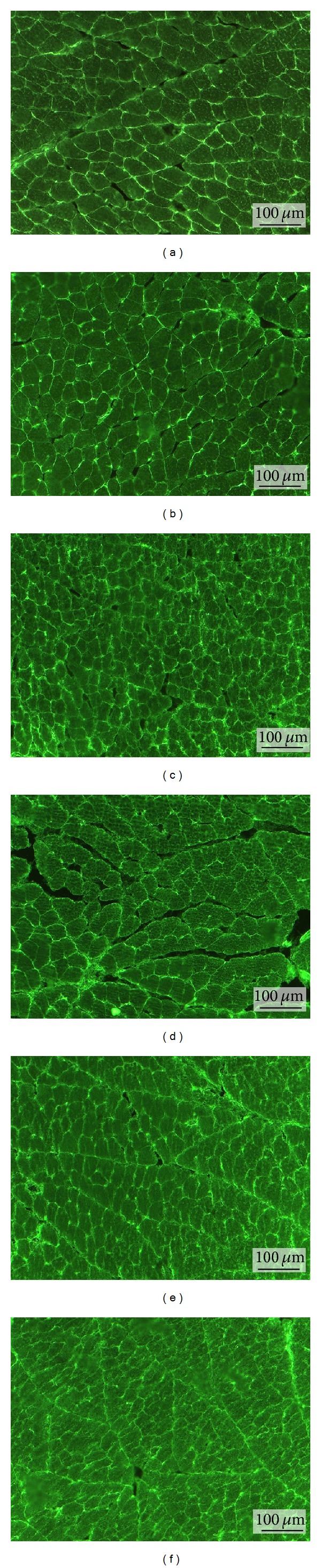
*In situ* zymography of the contralateral (a, c, and e) and ipsilateral (b, d, and f) masseter muscle. (a, b) Control group. (c, d) Experimental group 14 days after exodontia. (e, f) Experimental group 26 days after exodontia.

**Figure 4:**
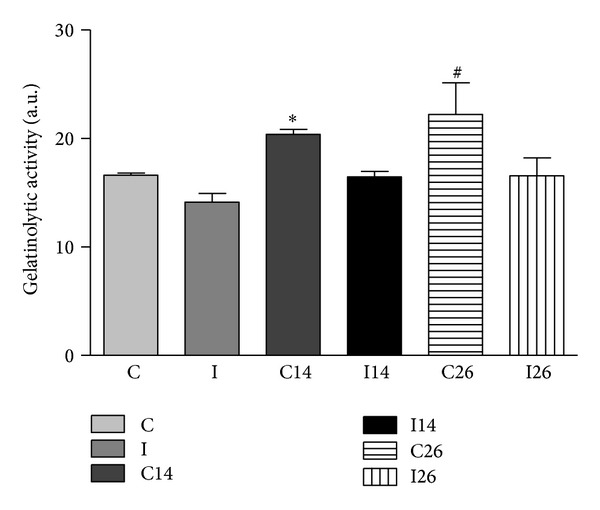
Quantitative analysis of the gelatinolytic activity found in muscle sections. Data are shown as the mean ± SD. *n* = 10 per group. *P < 0.05 C versus C14. ^#^ *P* < 0.05 C versus C26.

The* in situ *zymography allowed identification of the gelatinolytic activity, due to expression of MMPs on frozen sections of unfixed masseter muscle samples using a fluorogenic substrate (gelatine). Specific inhibitors for the different classes of proteinases were used to specify the gelatinolytic activity of the metalloproteinases. The sections were examined by fluorescent microscopy and the images were captured at 200x magnification (Figure 3). The proteolytic activity was detected as bright green fluorescence and was identified in the endomysium and perimysium and around the blood vessels in both exodontia side and contralateral side muscles of all three analyzed groups. Quantification of the proteolytic activity was showed on all deep masseter muscles of control group and after 14 and 26 days of extraction of left upper molar groups (Figure 4). A statistical difference was observed when the contralateral muscles (14 and 26 days) were compared to the control group (*P* < 0.05). Phenanthroline at 10 *μ*Mol/L inhibited the proteolysis completely, confirming that metalloproteinase was responsible for the protease activity.

The data distribution of gelatinolytic activity in muscle sections was not normal. Thus, a nonparametric statistical analysis was used, that is, Kruskal-Wallis test followed by Dunn's Multiple Comparisons.

### 3.4. Immunohistochemistry

The distribution of MMPs and TIMPs on the masseter muscle was evaluated in the experimental group after 14 and 26 days after tooth extraction and in the control group.

**Figure 5:**
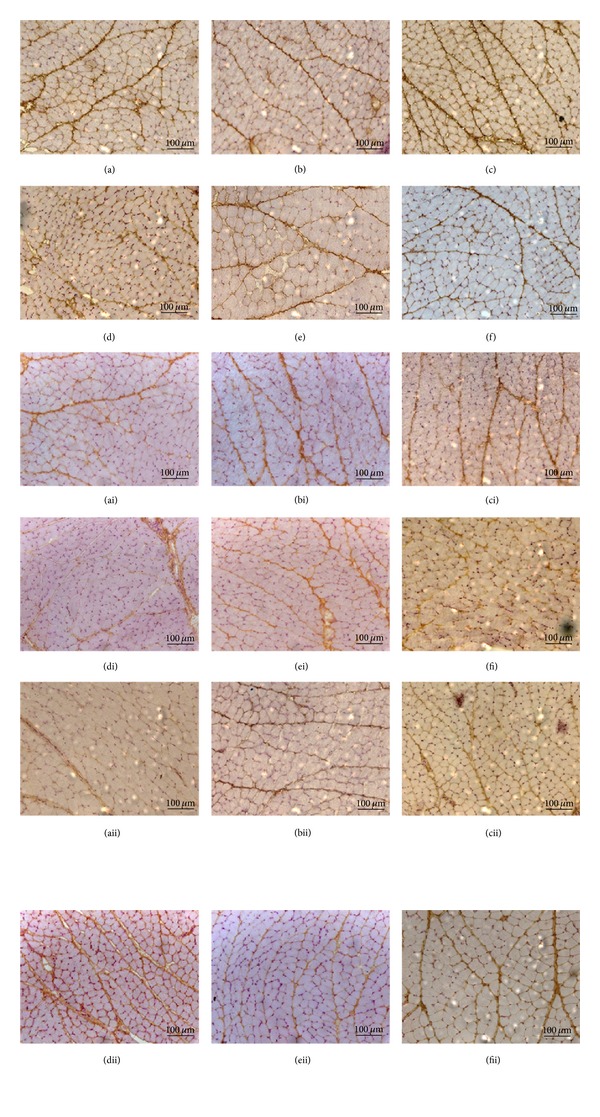
Immunolocalization of MMP-2 in the masseter muscle ((a) contralateral control, (b) ipsilateral control, (c) 14 days contralateral, (d) 14 days ipsilateral, (e) 26 days contralateral, and (f) 26 days ipsilateral), MMP-9 ((ai) contralateral control, (bi) ipsilateral control, (ci) 14 days contralateral, (di) 14 days ipsilateral, (ei) 26 days contralateral, and (fi) 26 days ipsilateral), and MMP-14 ((aii) contralateral control, (bii) ipsilateral control, (cii) 14 days contralateral, (dii) 14 days ipsilateral, (eii) 26 days contralateral, and (fii) 26 days ipsilateral).

The immunohistochemistry for MMPs demonstrated an increased expression of MMP-2 in the masseter muscle compared to MMP-9 and MMP-14. MMP-9 and MMP-14 were found only in the perimysium, while MMP-2 showed intense staining in both the endomysium and perimysium (Figure 5).

**Figure 6:**
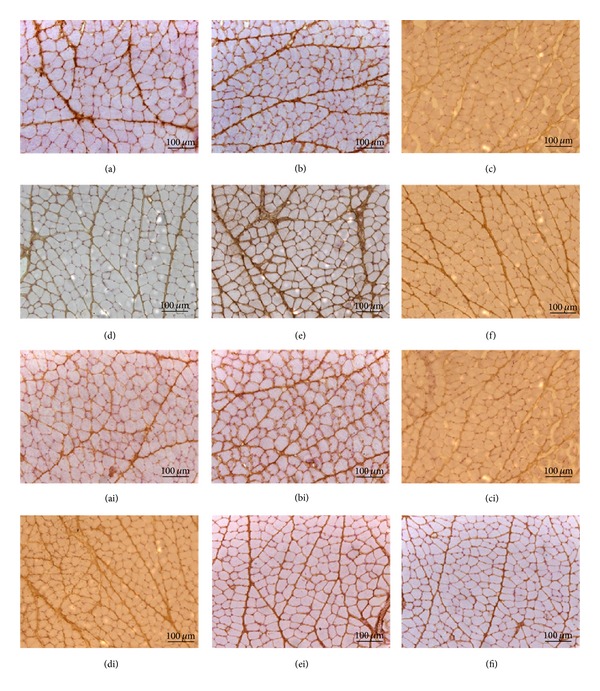
Immunolocalization of TIMP-1 in the masseter muscle ((a) contralateral control, (b) ipsilateral control, (c) 14 days contralateral, (d) 14 days ipsilateral, (e) 26 days contralateral, and (f) 26 days ipsilateral) and TIMP-2 ((ai) contralateral control, (bi) ipsilateral control, (ci) 14 days contralateral, (di) 14 days ipsilateral, (ei) 26 days contralateral, and (fi) 26 days ipsilateral).

**Figure 7:**
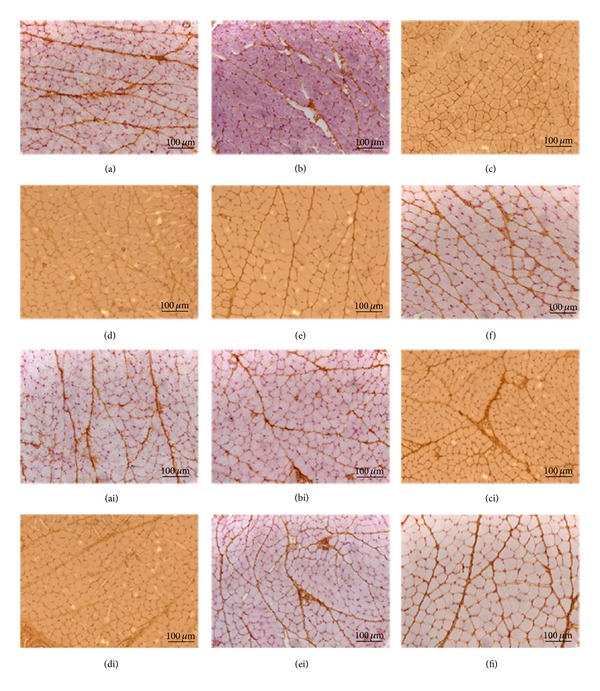
Immunolocalization of TIMP-3 in the masseter muscle ((a) contralateral control, (b) ipsilateral control, (c) 14 days contralateral, (d) 14 days ipsilateral, (e) 26 days contralateral, and (f) 26 days ipsilateral). Immunolocalization of TIMP-4 in the masseter muscle ((ai) contralateral control, (bi) ipsilateral control, (ci) 14 days contralateral, (di) 14 days ipsilateral, (ei) 26 days contralateral, and (fi) 26 days ipsilateral).

When comparing the different conditions, generally had lower intensity of staining for TIMPs on both sides of the masseter muscle after 14 days of extration. However, while TIMP-1 and TIMP-2 (Figure 6) intensely stained in both, the endomysium and perimysium, TIMP-3 and TIMP-4 had a marked staining only in the perimysium and only very faint staining in the endomysium (Figure 7).

## 4. Discussion

Microscopic analyses showed muscle fiber with flattened nuclei suggesting atrophic aspect on ipsilateral side of the exodontia and on contralateral side more rounded muscle fiber with some larger nuclei, after both 14 and 26 days of left molar teeth exodontia. The ipsilateral side of the molar teeth exodontia muscle fiber characteristic suggested reduced muscle tension and could result from less mechanical loading. This fact was different to the contralateral side muscle fiber characteristics, which revealed no atrophic effect induced by the same experimental model. According to Iyomasa et al. [21], it was reported that after unilateral molar extraction, the ipsilateral suprahyoid muscle showed atrophic aspects of subsarcolemmal and intermyofibrillar mitochondria. In addition, the same oclusal alteration performed in the present study showed morphological modifications in other structures of the stomatognatic system [22–24]. Then, the unilateral molar teeth exodontia induced different mechanical loading that made both sides of muscle fibers remodeling.

In nonfacial skeletal muscles, it is generally accepted that the remodeling process requires the activation of some enzymes known as matrix metalloproteinases (MMPs) [7, 8], which act on the extracellular matrix proteins (ECM). Extracellular matrix (ECM) placed in tendon tissue as well as peri- and intramuscularly ensures a functional link between the skeletal muscle cell and the bone [5]. Even though search about ECM response to mechanical loading and the mechanism in what manner it occurs on masticatory muscle are scarce, the changes in teeth occlusion may interfere in turnover of extracellular matrix (ECM).

The somewhat larger MMP-2 level in the exodontias side muscle suggests its participation in the atrophic muscle process and is according to Giannelli et al. and Skittone et al. findings that observed an increased expression of MMP-2 in muscle atrophic conditions [25, 26].

Several strategies have been studied for direct intervention in the activity of MMPs as a way to prevent pathological changes induced by them in some diseases, seeking to generate potent synthetic blockers of proteolytic activity. The identification of new topological allosteric sites of MMPs is a key challenge for the advancement in the development of more selective drugs. The connection to the active site specifically as well as the particular exosites of MMPs will improve the specificity and efficiency of inhibitors of MMPs [27]. Our gel zimography confirmed the matrix metalloproteinases-2 (pro-MMPs-2, 72 kDa) presence in all rat deep masseter muscles of the three groups: control and 14 and 26 days after unilateral exodontia. The levels of this enzyme showed tendency to increase in both ipsilateral sides of muscle after 14 and 26 days of unilateral extraction of left upper molar. The MMPs-2, 72 kDa, are synthesized as inactive zymogens in several cells [28] which are transported to the cell surface, where they became activated and either remain membrane-bound or are secreted into the extracellular space [29]. Thus, this fact suggested molecular muscle response to the inactivity, caused by the left side unilateral exodontia. In the contralateral side muscle, after 14 days, the level of pro-MMP-2, 72 kDa, also showed tendency to be higher than the control. While, in the same side, after 26 days, it showed a decreased tendency. This fact can be explained by adaptation of the contralateral muscle, after 26 days, despite a previous temporal study revealed that adaptation was not finished after 30 days, when analyzed in masseter muscle, using a splint in unilateral rat [30].

*In situ* zymography is an important method to reveal active MMPs in frozen and nonfixed tissue sections [31]. The proteolytic activation of the pro-MMPs seems to proceed via a stepwise mechanism. According to Matrisian [32], type 2 MMPs are associated with the function and dysfunction of skeletal muscle and play an important role in the degradation of type IV collagen, elastin, fibronectin, and laminin. Thus, the increase of active gelatinases detected on contralateral side muscles, in the experimental group (14 and 26 days), indicated a molecular response of the extracellular matrix. It could result from altered mechanical loading caused to counterbalance the left side unilateral exodontias.

In the present study, the immunolocalization for MMPs demonstrated that MMP-2 was expressed in the endomysium and perimysium; however, MMP-9 and MMP-14 stained intensely only in the perimysium, in both sides of the muscle of all groups. According to Lewis et al. [8] MMP-2 and MMP-9 (gelatinases), which degrade the basal lamina components, are the most widely evaluated MMPs in muscle tissues. An earlier study described the MMP-2 and MMP-9 proteins localization in the entire extracellular matrix of muscle, both around the fibers and capillaries, but MMP-2 was also present inside the skeletal muscle fibers [33]. These facts support Maskos and Bode [34] findings, that MMPs in rats masseter muscle although involved in degradation of extracelular matrix, also participate in other biologic processes.

The four types of tissue inhibitor metalloproteinases (TIMPs) are present in the masseter muscle. Although Gianelli et al. [25] and Rullman et al. [33] have described the presence of TIMPs in skeletal muscle, the current literature has very limited information about MMPs and their inhibitors (TIMPs) in masseter muscles.

Gomez et al. [16] and Cawston [35] described that besides inhibitory role, the TIMPs seem to have other functions, such as growth factor-like and antiangiogenic activity. TIMP-1 and TIMP-2 are capable of inhibiting activities of all MMPs with a preference for inhibiting MMP-2 and MMP-9, respectively [36]. According to Gianelli et al. [25] the MMP-2/TIMP-2 imbalance is one mechanism of muscle atrophy. Hunt et al. [3] emphasized that it is important to understand how muscles respond to clinical interventions to attain clinical success. Thus, it is extremely important to further expand the knowledge about the composition, organization, interaction, and molecular aspects of the extracellular matrix components because this knowledge may influence the modality of employed treatments.

## 5. Conclusion

Unilateral exodontias induced ipsilateral and contralateral side masseter muscle remodeling, as shown by histological characteristics, conventional zymography,* in situ* zymography, and immunohistochemistry. It was possible to conclude that this remodeling process requires the activation of MMPs and there was integrated participation of TIMPs. More studies are needed for a better understanding of the molecular participation in masticatory muscle remodeling, following environmental interventions such as functional appliance therapy or resulting from developmental situations.

Knowledge of MMPs and their association with tissue repair is an important step in the development of a new pharmacological class for the treatment of muscles diseases. The effects of pharmacotherapy inhibition of MMPs should be leveraged from the knowledge of the mechanisms involved and should be extrapolated to clinical trials for the safety of the proposed drug.
